# Rural Indian tribal communities: an emerging high-risk group for HIV/AIDS

**DOI:** 10.1186/1472-698X-5-1

**Published:** 2005-02-21

**Authors:** Eknath Naik, Arun Karpur, Richard Taylor, Balasubramaniam Ramaswami, Seetharam Ramachandra, Bindu Balasubramaniam, Sagar Galwankar, John Sinnott, Sarah Nabukera, Hamisu M Salihu

**Affiliations:** 1 Department of Epidemiology and Biostatistics, College of Public Health, University of South Florida, Tampa, Florida, USA; 2 Swami Vivekananda Youth Movement, Mysore, Karnataka, India; 3 Division of Infectious Diseases, Department of Internal Medicine, University of South Florida, Tampa, Florida, USA; 4 Department of Maternal & Child Health, School of Public Health, University of Alabama at Birmingham, USA

## Abstract

**Background:**

Rural Indian tribes are anthropologically distinct with unique cultures, traditions and practices. Over the years, displacement and rapid acculturation of this population has led to dramatic changes in their socio-cultural and value systems. Due to a poor health infrastructure, high levels of poverty and ignorance, these communities are highly vulnerable to various health problems, especially, communicable diseases including HIV/AIDS. Our study sought to assess knowledge, attitudes and practices regarding sexuality, and the risk factors associated with the spread of HIV/AIDS and STDs among these communities.

**Methods:**

A nested cross sectional study was undertaken as part of the on going Reproductive and Child Health Survey. A total of 5,690 participants age 18–44 were recruited for this study. Data were obtained through home interviews, and focused on socio-demographics, knowledge, attitudes and behaviors regarding sexuality, HIV/AIDS and other STDs.

**Results:**

The study revealed that only 22% of adults had even heard of AIDS, and 18 % knew how it is transmitted. In addition, only 5% knew that STDs and AIDS were related to each other. AIDS awareness among women was lower compared to men (14% vs.30 %). Regarding sexual practices, 35% of the respondents reported having had extramarital sexual encounters, with more males than females reporting extramarital affairs.

**Conclusion:**

Lack of awareness, permissiveness of tribal societies for premarital or extra-marital sexual relationships, and sexual mixing patterns predispose these communities to HIV/AIDS and STD infections. There is a dire need for targeted interventions in order to curtail the increasing threat of HIV and other STDs among these vulnerable populations.

## Background

India is the second most populous nation in the world and has changing sociopolitical and demographic characteristics as well as varied morbidity and mortality patterns [1]. These changes, in conjunction with the country's high population growth rate, have exacerbated the prevailing and emerging public health challenges the country is facing. Since 1986 when the first case of human immunodeficiency virus (HIV) was reported in India [2], it has become imperative to include acquired immunodeficiency syndrome (AIDS) on its long list of public health issues that need to be addressed.

As a direct result of these challenges, India has begun to assess and monitor the impact of HIV/AIDS throughout the country's various states and regions with the assistance of several international health organizations. According to 2003 estimates from UNAIDS, approximately 5.1 million individuals in India are infected with the HIV virus [3]. Furthermore, recent studies indicate that transmission of HIV is no longer confined to high-risk urban populations, but is spreading across rural settings as well [4]. This trend is a cause for concern as AIDS is increasingly hampering social and economic development throughout the country.

For effective control of the spread of HIV/AIDS, it is crucial to have data on knowledge, attitudes and behavioral practices for specific population as research has shown that socio-cultural influences, traditional lifestyles, societal norms, and traditions influence HIV/AIDS transmission rates [5,6]. Because India's HIV/AIDS transmission pattern is predominantly heterosexual (85% of all newly reported cases) [7], subcultures that have relaxed marital structures or are tolerant of high-risk sexual practices (e.g., sex with a commercial sex worker) are particularly vulnerable to the spread of HIV/AIDS and STDs within their communities [7,8]. With more Indian men reporting premarital and extramarital sexual activity, women who marry as teenagers are vulnerable to HIV/AIDS infection and STDs [9,10].

The rapid spread of HIV/AIDS in rural Indian communities has been attributed to the country's poor health infrastructure, poverty and lack of awareness [4,10]. Despite these indicators, little is known about the risk factors, transmission rates, or the impact AIDS will have in these areas in the future. Traditionally, there has been little research and only a paucity of health-related research conducted among this potentially high-risk vulnerable population.

Throughout India, approximately 8% of the population lives within rural tribal communities, which are collectively referred to as 'Tribes'. These communities are geographically distinct; with each tribe having its own unique customs, traditions, beliefs and practices. Even within a particular tribal entity, differences in dialect, health practices, unique customs, values, and traditions are apparent. In rural Indian communities indices of reproductive health are typically very poor: maternal mortality rate is about 230 per 100,000 live births and 61.2% of the women suffer from at least one gynecologic pathology [11]. Because tribal groups have existed on the fringe of Indian society, they may still be unaware or indifferent to the potential health threats from HIV/AIDS. Ascertaining whether or not tribal communities are potentially a high risk group warranting intervention is a necessary step in India's war on AIDS. Accordingly, we undertook this study to explore the risks for this special group of people.

## Methods

We conducted a cross-sectional study nested within an existing enumerative study referred to as the Reproductive and Child Health Survey (RCHS). The RCHS was initially designed to enumerate and ascertain basic demographic and health profiles for all tribal members. Data collection for this particular study was done in two phases. Phase one involved adding additional questions to the original RCHS study to assess risk factors (knowledge, attitudes and behavioral practices) associated with the transmission of HIV/AIDS and other communicable diseases.

### Study population

**Figure 1:**
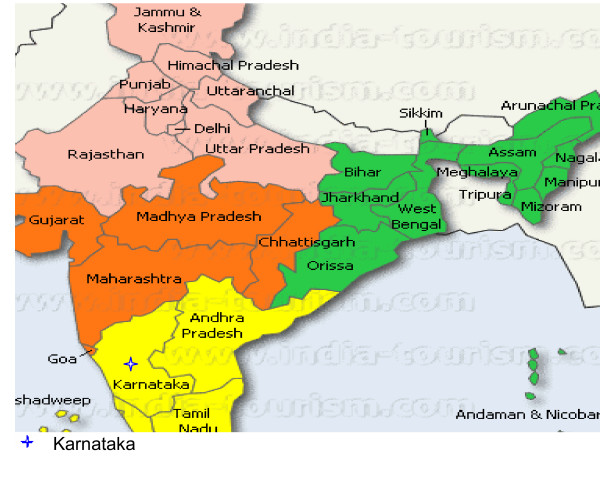
Map of India showing the region of Karnataka where the study was conducted. Used with permission from India-tourism.com

**Figure 2:**
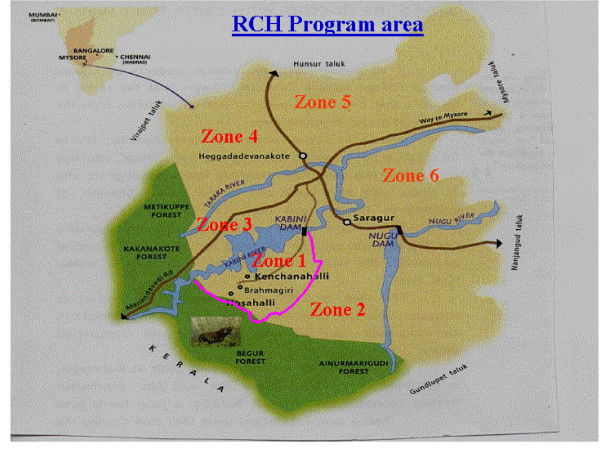
The reproductive and child health program area in the southern part of Karnataka. Used with permission from the Reproductive Child Health Survey (RCHS) project

The study population comprised of tribal communities living in the southern region of Karnataka (Figures 1,2). Members of these tribes have traditionally derived much of their livelihood from the country's vast reserve of natural forest resources. However over the years, these tribes have been forced to migrate from their ancestral land and are currently living within poor rural communities throughout the state. The initial displacement was as result of the submergence of their traditional homelands through the construction of the Kabini dam, and the second displacement was as a result of 'Project Tiger,' a wildlife conservation project that displaced them to their current location in the southern region of Karnataka, where this study was conducted.

### Sampling

Because of the enumerative nature of the RCHS, all persons within the age group 18–44 years who participated in the RCHS study were included for phase one of this study. This age group was selected as the focus of the initial study was on reproductive health issues.

### Survey instruments

A semi-structured questionnaire with both open- and close-ended questions was developed to collect information on knowledge, attitudes and behaviors regarding HIV/AIDS, as well as other relevant demographic information not included in the RCHS. The instrument was developed in English, translated to the native language, and subsequently back translated to English for content and language verification. The survey instrument was field-tested for validity purposes and modified accordingly.

### Data collection

A team of ten interviewers from the local tribal communities with a minimum of high school education were selected and trained for two weeks to ensure uniform and high-quality data collection. All adults were interviewed separately to ensure confidentiality. Each interviewer read out each of the questions and response choices (where appropriate) to the interviewees and recorded all answers directly on the questionnaire. Verbal informed consent was obtained from each respondent prior to starting the interview.

### Data analysis

Data were entered into an electronic database using Sybase Central Software (Sybase, Inc. Dublin, CA). To ensure confidentiality, all respondent identifiers were expunged to create a secondary data set that was used for the final analysis. Frequency tables were generated for selected demographics and health related categorical variables. In addition, univariate analysis was performed on relevant continuous variables. The findings are presented below.

### Ethical consideration

Ethical clearance was obtained from ethical boards within each of the tribal communities, and appropriate government agencies were informed about the study objectives. Permission was also obtained from the Institutional Review Board (IRB) at the University of South Florida in Tampa, Florida. Verbal informed consent was obtained from the local leaders, and the individual participants. Because of the high levels of illiteracy, it was not feasible for us to obtain written consent.

## Results

### Demographic profile

A total of 11,379 individuals from the 5 tribal communities had been enumerated as part of the RCHS. Of these, 5,690 were within the study age range (18–44 years) and formed the basis for this analysis.

Table 1 shows the demographic profile of the study participants. The mean age of the study group was 31 years. There were more males than females; 53% vs.47%. Eighty four percent were married (91% females & 78% males). The average age at marriage was 13 years for females, and 22 years for males. Only 28% (27% female, & 30% male) of the population was literate i.e. able to read and write in any of the Indian languages. The majority of respondents (67 %) reported living in tiled roof houses with mud flooring, while only 40 % indicated easy access to potable water. Agriculture was the major source of income in these communities. The reported average daily income ranged from US $1.50 to $2.00. Approximately 35% of the respondents migrated on average three to four months each year to nearby areas for work.

**Table 1:** Demographic profile of the study participants

Characteristics	Number (%) (N = 5,690)
Mean age	31 years

Sex	
Men	3,016 (53)

Women	2,674 (47)

Currently living in tiled-roof housing	3,812 (67)

Access to potable water	2,276 (40)

	
Migrated to find work	1,992 (35)

Currently married	
Men	2,353 (78)*
Women	2,433 (91)**
All	4,786 (84)

Literacy***	
Men	814 (27)*
Women	802 (30)**
All	1,616 (28)

Mean age of first marriage	Mean years (95% CI)
Men	22.0 (19.5–24.5)
Women	13.0 (11.2–14.7)
All	15.0 (11.2–18.8)

*Percentages are based on the total number of men in the study.

**Percentages are based on the total number of women in the study.

***Literacy as defined by census operations of India is the ability to read and write in any of the Indian languages.

CI = Confidence interval

### Unique sexual practices among tribal members

The findings revealed that these tribal communities did not have a structured marital system; instead members practiced a form of serial monogamy in which they change partners and remarry every four to five years. Regarding sexual practices, 35% of the respondents reported either premarital affairs or extramarital affairs (Table 2). However such practices were more common in men compared to women. Furthermore, 20% of the male participants reported having had sex with a commercial sex worker (CSW) during the period the wife had had a child.

**Table 2:** Self reported sexual practices of respondents

Characteristics	N = 5,690	
Age at first sexual activity	Mean years (95% CI)	
Men	17.0 (13.4–20.6)	
Women	13.0 (11.5–14.5)	
Premarital or extramarital sexual encounters	Number (n)	(%)
Males	1,434	72.0
Females	558	28.0
Total	1,992	35*
Sex with commercial sex worker within a year after spouse giving birth (men only n = 3,016)	470	20

* Percent based on the total sample of 5,690 respondents

### Knowledge and beliefs about HIV/AIDS and STDs

Among these communities, there was a low level of knowledge on HIV/AIDS; only 22 % of all study participants (n = 1,252) had heard of AIDS (Table 3). Among those who have heard of AIDS, less than 20 % (n = 250) knew how HIV/AIDS was transmitted (16.8 % male vs. 8% females). About 98 % were not aware of the methods to prevent HIV/AIDS transmission. As many as 30 % (n = 376) of those who had heard of AIDS believed that "sinners" will get AIDS, while 10 % (n = 125) believed that AIDS and STDs could be prevented by the sterilization of women. Fifteen percent (n = 188) thought "AIDS is acquired by looking at a person who has AIDS," and 18 % (n = 225) believed that "AIDS is acquired by talking to a person who has AIDS." Only 5 % knew that a relationship exists between HIV/AIDS and STDs. Interestingly, 4 percent (n = 51) believed that there was a cure for AIDS. Most had not heard of STDs, and of those who had heard of them only 1 percent (n = 16) were aware of associated symptoms.

**Table 3:** Knowledge and beliefs about HIV/AIDS and STDs

Knowledge and beliefs about HIV/AIDS	No. of study subjects N = 5,690 No. of study subjects who have ever heard of HIV/AIDS N = 1,252		
	N	Percent of 5,690	Percent of 1,252

Have ever heard of HIV/AIDS	1,252	22.0	100.0
Know how HIV/AIDS is transmitted	250	4.4	20.0
Know methods to prevent the transmission of HIV/AIDS	114	2.0	9.1
Believe "sinners" will get AIDS	376	6.6	30.0
Believe AIDS and STDs can be prevented by sterilization of women	125	2.2	10.0
Believe AIDS is acquired by looking at an infected person with AIDS	188	3.3	15.0
Believe AIDS is acquired by talking to a person who has AIDS	225	4.0	18.0
Believe there is a cure for HIV/AIDS	51	0.9	4.1

## Discussion

In today's modern world, it is difficult to imagine societies that are still socially and culturally isolated from the rest of civilization; however, they do exist. The tribal societies throughout India have remained socially and culturally alienated from mainstream Indian society until developmental and conservation activities in tribal areas forced interactions between them. Displacement of the tribal people of southern Karnataka has led to a complex process of rapid acculturation and loss of cultural identity; as they struggle to maintain their traditional social structure, they must adopt new skills, beliefs, and practices necessary for success in their new environment. During this acculturation process, they have been faced with a myriad of public health challenges complicated by poverty, ignorance, and reluctance to abandon traditional beliefs and practices that would allow them to assimilate successfully.

To date this is the first health related study among the displaced tribal communities of southern Karnataka that has attempted to assess risk threshold for the transmission and spread of HIV/AIDS and other STDs. It is not surprising that knowledge and awareness about HIV/AIDS and STDs was very low among tribal communities compared to the national figures given the degree of isolation, low literacy rates, and minimal access to information.

The high level of poverty, inadequate health resources, ignorance and high-risk beliefs and practices among the tribal communities has contributed to the vulnerability of this population. As such it has created a highly susceptible population for the rapid spread of HIV/AIDS and other STDs' as well.

Because tribal members are forced to migrate outside of their communities in search for work and increased wages, this may contribute to the spread of HIV/AIDS as many engage in extramarital affairs, seek commercial sex partners, or are under the threat of sexual harassment (females). While there has been limited scientific research exploring the cultural context of extramarital sexual behaviors, it is generally noted that in these communities, extramarital affairs are condoned and widely practiced especially during periods when women are pregnant or nursing or during period of travel for work [8]. This kind of behavior creates a fertile ground for HIV transmission and spread. Our data indicate that tribal women are particularly vulnerable for HIV/AIDS in this population since many of them commence sexual activity at an early age, and get married early as well. Also, they are in a culture that condones extramarital sex, and this exposes the women to a particularly precarious situation, increasing their risk for acquiring HIV.

## Conclusion

It is evident from this study that the Indian tribal community is experiencing a latent phase that is potentially a precursor for an HIV/AIDS epidemic. There is a high prevalence of behavioral risk factors, coupled with ignorance, and inadequate health infrastructure thus creating a potential risk for rapid spread of HIV/AIDS, as well as other related diseases. In a country that is struggling to contain the spread of HIV, it is particularly important for concerned parties to pay attention to this population. Currently, virtually no resources are allocated toward the treatment of those infected with HIV/AIDS; the main stay of management is through education and preventive measures to control the spread of the scourge as they represent the most practical and cost effective strategies in this developing nation. It therefore becomes imperative and urgent to address the health concerns revealed in our study in order to formulate effective, culture-sensitive and appropriate intervention programs so that an imminent disaster (i.e. HIV/AIDS epidemic) in this remote and isolated communities can be averted.

## Competing interests

The author(s) declare that they have no competing interests.

## Authors' contributions

All the authors were involved in the design of the study, analysis, interpretation and development of the manuscript.

## Pre-publication history

The pre-publication history for this paper can be accessed here:


